# Lipidomic Landscapes of Cryopreserved Sperm from Alpine and Spanish–Creole Bucks

**DOI:** 10.3390/ani15131897

**Published:** 2025-06-27

**Authors:** Mustafa Bodu, Mustafa Hitit, Selamawit Woldesenbet, Muhammet Raşit Uğur, Zeynep Erdoğan, Olivia Chika Greenwood, Raheem Davian Murray, Andres Pech Cervantes, Erdoğan Memili

**Affiliations:** 1 Cooperative Agricultural Research Center, College of Agriculture, Food, and Natural Resources, Prairie View A&M University, Prairie View, TX 77446, USA; mbodu@selcuk.edu.tr (M.B.); muhitit@pvamu.edu (M.H.); sewoldesenbet@pvamu.edu (S.W.); vh.zeyneperdogan@gmail.com (Z.E.); ogreenwood@pvamu.edu (O.C.G.); rmurray13@pvamu.edu (R.D.M.); aapechcervantes@pvamu.edu (A.P.C.); 2 Department of Reproduction and Artificial Insemination, Faculty of Veterinary Medicine, Selçuk University, Konya 42075, Türkiye; 3 IVF Michigan Fertility Centers, 37000 Woodward Ave #350, Bloomfield Hills, MI 48304, USA; rugur@ivf-mi.com

**Keywords:** Alpine, Spanish–Creole, goat, sperm, lipidomics

## Abstract

This study provides a comparative analysis of the post-thaw sperm lipidomic profiles of Alpine and Spanish–Creole goat breeds to explore breed-specific differences in fatty acid composition and their implications for sperm function and reproductive efficiency. Lipids were extracted from cryopreserved semen samples of Alpine (n = 7) and Spanish–Creole (n = 4) mature bucks and subsequently analyzed by gas chromatography–mass spectrometry (GC-MS), with 21 fatty acids identified within the two breeds. Eight of these fatty acids, namely 13:0, 16:0, 18:0, 24:0, 14:1, 18:1 (cis-9), 24:1, and 18:2 showed significant differences (*p* < 0.05). The levels of 16:0, 18:0, 24:0, 18:1 (cis-9), 18:1, and 18:2 were higher in the Alpine breed, whereas the levels of 13:0, 14:1, and 24:1 were higher in the Spanish–Creole breed (*p* < 0.05). Of those, 16:0, 18:1 (cis-9), and 18:2 were both statistically and biologically significant (*p* < 0.05, FC > 2). Concentrations of the total fatty acids, total saturated fatty acids (Total-SFA), and total polyunsaturated fatty acids (Total-PUFA) were significantly higher in the Alpine breed, whereas the concentrations of the total cis-monounsaturated fatty acid (Total cis-MUFA) were significantly higher in the Spanish–Creole breed (*p* < 0.05). Network and pathway analyses revealed that 16:0, 18:1 (cis-9), and 18:2 contributed to the most central nodes of the lipidomic network, which may support membrane stability and cryotolerance. The lipidomic differences observed between breeds may be attributed to both genetic and environmental factors and may provide valuable tools for enhancing breeding strategies, artificial insemination programs, and sperm cryopreservation techniques.

## Simple Summary

This study was aimed at comparing the post-thaw sperm lipidomic profiles of Alpine and Spanish–Creole goat breeds. Lipids were extracted from cryopreserved semen samples of Alpine (n = 7) and Spanish–Creole (n = 4) mature bucks, and analyzed by gas chromatography–mass spectrometry (GC-MS). A total of 21 fatty acids were identified. Eight of these fatty acids, 13:0, 16:0, 18:0, 24:0, 14:1, 18:1 (cis-9), 24:1, and 18:2 showed statistically significant differences (*p* < 0.05). Of those, 16:0, 18:1 (cis-9), and 18:2 were both statistically and biologically significant (*p* < 0.05, FC > 2). Network and pathway analyses revealed that 16:0, 18:1 (cis-9), and 18:2 are the key components influencing the lipidomic network’s central nodes, which promote membrane stability and cryotolerance. Variations in sperm lipid profiles between breeds might stem from genetic and environmental factors, offering valuable insights for enhancing sperm cryopreservation techniques, breeding strategies, and artificial insemination programs.

## 1. Introduction

Enhancing reproductive efficiency contributes not only to global food security but also to the sustainability of livestock production systems. Goats serve as a critical resource in many regions worldwide, supplying meat, milk, and fiber [1]. The integration of reproductive biotechnologies and genetic improvement programs targeting reproductive traits in goats can enhance productivity, support population expansion, and maintain genetic diversity in food-producing animals [2,3]. High sperm quality, assessed by parameters such as motility, viability, and morphology, is closely associated with successful fertilization, reproductive success, and offspring health [4,5]. Among the many contributors to sperm function, lipid composition of the sperm membrane plays a central role, as lipids influence motility, membrane fluidity, and provide protection against oxidative damage, thereby supporting fertilization potential [6,7,8].

Sperm traits such as motility, morphology, and viability are closely associated with fertilization success and overall reproductive efficiency. These characteristics are influenced by both the structural components of sperm (particularly membrane lipids) and the functional properties essential for fertilization [9,10]. Strong correlations have been established between sperm quality and fertility outcomes across different livestock species [11,12,13]. In recent years, advances in sperm lipidomics have provided valuable insights into the roles of lipids in sperm membrane functionality and fertility [14]. In goats, specific lipid species have been linked to improved motility and enhanced reproductive success, highlighting the importance of lipid metabolism in sperm function [15,16].

Lipid profiles are also associated with important reproductive parameters, including cryopreservation success and conception rate [17]. More specifically, researchers have indicated that infertile men have a higher mean AA:DHA ratio and AA:EPA than fertile men [18]. Docosahexaenoic acid (DHA) is a long-chain polyunsaturated fatty acid that is a critical factor for spermatogenesis and sperm maturation. Interestingly, increased sperm DHA content has been positively correlated with fertilization ability [19]. While research on lipidomics in small ruminants, particularly goats, remains limited [20], significant progress has been made in studying lipid metabolism in large livestock species such as cattle [21] and pigs [22]. This indicates a considerable knowledge gap that can help elucidate biological mechanisms contributing to reproductive performance [8]. Overall, the varying lipidomic profiles in sperm across goat breeds may facilitate breed-specific reproductive strategies. These profiles may account for differences in fertility rates and reproductive efficiency, thereby improving productivity and health in goat populations [21].

Lipidomics is a compelling approach used to analyze the lipid profiles of cells, including sperm, and to identify markers that may influence sperm function and male reproductive health [23,24]. Several lipid classes, such as phosphatidylethanolamines, phosphatidylcholines, cholesterol, and triglycerides, have been identified as relevant to sperm function and can be further characterized using advanced techniques, including mass spectrometry [25]. Despite the importance of elucidating how lipid profiles associate with reproductive performance, fertility, and ultimately with herd productivity, there are relatively few data available on differences in sperm lipidomic markers specifically attributable to breed. Lipids such as diacylglycerol [26] and plasmalogens [27] are essential for maintaining sperm membrane integrity and improving cryopreservation outcomes. These lipids contribute to membrane fluidity and resistance to freezing-induced damage, thereby supporting the success of artificial insemination protocols [28]. Environmental factors such as temperature and nutritional status may influence sperm lipid composition through molecular mechanisms, offering non-genetic approaches to enhance fertility [29]. This study represents a novel contribution by investigating the fatty acid profiles of goat sperm, addressing a knowledge gap in reproductive biology. Identifying breed-specific lipid signatures may improve fertility outcomes, increase herd productivity, and contribute to sustainable animal production and food security efforts [21,30].

The objective of this study was to elucidate a comparative analysis of sperm lipidomes between Alpine and Spanish–Creole bucks using gas chromatography–mass spectrophotometry (GC-MS). The findings of this study allow us to identify the signature of lipidomic profiles based on different breeds of goats and use the new knowledge for applied reproductive biology.

## 2. Materials and Methods

### 2.1. Study Design, Sample Preparation, Cryopreservation, and Determination of Post-Thaw Semen Parameters

Semen samples were collected from two goat breeds, Alpine (n = 7) and Spanish–Creole (n = 4), twice a week with an artificial vagina between August and October 2024 at the International Goat Research Center in the College of Agriculture, Food, and Natural Resources at Prairie View A&M University. All bucks were housed under identical management conditions at the research facility. Environmental variables such as diet composition, stress levels, and photoperiod were strictly controlled to ensure uniformity throughout the experimental period. Cryopreservation was performed using the same extender (Andromed, Minitube, Verona, WI, USA) for all sperm samples according to a modified protocol of [31,32,33]. The semen samples were collected with an artificial vagina, and diluted in the extender at 37 °C to a final concentration of approximately 200 × 10^6^ sperm per straw. Sperm samples in extenders were then loaded into 0.25 mL French straws, equilibrated at 4 °C for 3 h, and frozen in liquid nitrogen vapor (~−100 °C) for 15 min before being stored in liquid nitrogen at −196 °C. After completing the cryopreservations, samples were thawed at 37 °C in the water bath for 30 s. Motility and other kinematic parameters (total motility, progressive motility, VAP, VCL, VSL, ALH, BCF, LIN, STR, AREA, WOB, and ELONGATION) have been measured (Hamilton Thorne IVOS^®^ II, Beverly, MA, USA). Semen samples that had >60% were included in the lipid extraction according to total motility results [34].

### 2.2. Extraction of Lipids from Sperm Samples

#### 2.2.1. Sample Preparation

Semen samples were washed according to a modified protocol [35]. Samples containing 200 × 10^6^ sperm/mL were centrifuged at 3000× *g* at 4 °C for 10 min. The supernatant (extender and seminal plasma) was discarded. After the addition of 1.5 mL PBS, samples were centrifuged at 3000× *g* at 4 °C for 10 min, and the supernatant was again discarded. An additional 1.5 mL of PBS was added to the pellet, and the sample was sonicated at 50 amplitudes for 10 s (Qsonica, #Q700, Qsonica L.L.C., Newtown, CT, USA). Following a final centrifugation at 15,000× *g* at 4 °C for 10 min, the supernatant was discarded, and the sample was immediately processed for lipid extraction.

#### 2.2.2. Lipid Extraction Protocol

Lipid extraction was performed according to a modified protocol [14,35]. Lipids were extracted using a chloroform–methanol (2:1) solution. Cold chloroform (1 mL at 4 °C) and methanol (0.5 mL at 4 °C) were added to the sperm cell pellet. The mixture was vortexed thoroughly and incubated for 10 min at room temperature (RT) to dissolve the lipids. The sample was vortexed continuously for 1 h to maximize extraction. Subsequently, 0.2 mL distilled water was added, and the sample was vortexed for another 30 s before being centrifuged at 15,000× *g* at 4 °C for 10 min to separate the two phases. The extracted lipids were contained in the lower chloroform phase and pipetted to a clean tube. Chloroform was evaporated using a rotary evaporator (Eppendorf Vacufuge, 5305 Plus, Enfield, CT, USA) at 45 °C for 45 min. The dried lipids were stored at −20 °C until further analysis.

#### 2.2.3. Preparation of Internal and External Standards

The internal standard, methyl nonadecanoate (Nu-Check Prep, Inc., Catalog No. N-19 M, Elysian, MN, USA) dissolved in 20 mL of hexane, was prepared and diluted to 200 µg/mL as a working solution. Unknown samples were spiked with 10, 20, 50, and 100 µg of the internal standard to establish the best internal standard concentrations. After observing the results, the most appropriate concentration was 20 µg. The FAME mix C4-C24 (Sigma-Merck, Cat No. 18919, St. Louis, MO, USA) was utilized as an external standard. Dilutions were then made using 0, 10, 50, 100, 200, and 400 µg/mL of external standards, and each was spiked with 20 µg of the internal standard, and a calibration curve was created (Supplementary Figure S1).

#### 2.2.4. Preparation of Fatty Acid Methyl Esters (FAME)

Fatty acid methylation was performed according to the method described in [36]. After chloroform evaporation, 850 µL of hexane was added to dried lipids, and the mixture was transferred into 7.5 mL glass vials. Then, 250 µL of methanol mixed with 0.33 M NaOH was added to catalyze the reaction. The vials were tightly sealed, covered with parafilm, and topped with aluminum foil. The samples were heated at 60 °C on a shaker (Thermo Scientific MAXQ 4450, Waltham, MA, USA) for 90 min. Once the reaction was complete, the samples were cooled and incubated at RT overnight to allow phase separation. For analysis, the upper hexane phase was transferred into 1.5 mL GC vials, and then 20 µg of internal standard was added to each of the unknown samples before performing the gas chromatography.

#### 2.2.5. Gas Chromatography–Mass Spectrophotometry (GC-MS) Analysis

Gas chromatography–mass spectrometry analysis was conducted using an Agilent 7890A GC System (Agilent Technologies, Santa Clara, CA, USA) coupled to an Agilent 5975C inert XL MSD with the triple-axis mass detector, an Agilent 7693 Series Autosampler, and an HP-5ms GC capillary column (30 m × 0.25 mm i.d. × 0.25 μm film thickness; J&W Scientific, Folsom, CA, USA). The helium carrier gas had a constant flow rate of 1 mL/min. An injection mode of spitless was employed with an injection volume of 1 µL, which was injected into the inlet heated at 250 °C. A standard septum purge was carried out after the sample injection at 3 mL/min, and the helium carrier gas had a constant flow rate of 1 mL/min. The transfer line, ion source, and quadrupole were heated at 280 °C, 230 °C, and 150 °C, respectively. The initial temperature of the oven was programmed at 80 °C, with a hold time of 0.5 min, followed by 30 °C/min to 200 °C, 2 °C/min to 230 °C, a hold for 3 min, then 20 °C/min to 260 °C, and then a final 5 min maintenance at 260 °C. The total run was 29 min. Ionization was achieved via electron impact (EI) at 70 eV. Full scan mode (*m*/*z* 50–700) was used with an acquisition rate of 2.3 scans/s in the MS setting. Agilent Masshunter (version 10.2) software was used for data acquisition and processing. Peaks were identified based on retention times of the standards, and relative proportions of each fatty acid were calculated. The results were reported as ng/mL per sample. The normalized quantity of each fatty acid was calculated by dividing the gravimetric concentration by the total fatty acid concentration. The saturation index (SI) was calculated as the quantity of total SFA to total UFA (including cis-MUFA, trans-MUFA, and PUFA) concentration [14]

### 2.3. Statistical Analysis

Univariate and multivariate analyses were conducted to evaluate the association between fatty acid abundance and goat breeds. The statistical analyses were performed using MetaboAnalyst 6.0 (https://www.metaboanalyst.ca/, accessed on 8 January 2025) [37]. For multivariate analyses, the data were scaled and normalized using the “auto scale” option, in which the mean value of variables was subtracted from the data point, and the results were divided by the standard deviation of that variable. Partial Least Squares Discriminant Analysis (PLS-DA) was performed to identify classification between groups, and the contribution of each variable to the PLS-DA model was determined by calculating the variable importance in projection (VIP) score. For univariate analyses, a simple unpaired t-test was performed to determine the statistical significance between the Alpine and Spanish–Creole breeds, and statistical significance was set at *p* < 0.05, based on the conventional biological and statistical criteria. An unpaired fold change (FC) analysis was performed to compare the absolute values of the two group means. A threshold of FC ≤ 0.5 or FC ≥ 2.0 was used to identify biologically relevant differences. Fold change values were calculated using the raw (non-normalized) data to ensure accuracy. Pearson’s correlation was used to assess the relationships among fatty acids using individual animal data. A correlation network was then constructed based on Debiased Sparse Partial Correlation (DSPC) analysis [38].

## 3. Results

Motility and kinematic parameters were evaluated in post-thaw sperm samples. Among these, beat cross frequency (BCF) was significantly higher in the Alpine group compared to the Spanish–Creole group (33.29 ± 0.88 Hz vs. 29.67 ± 1.11 Hz; *p* = 0.017). Additionally, AREA was significantly lower in Alpine bucks than in Spanish–Creole bucks (17.65 ± 0.11 µm^2^ vs. 18.48 ± 0.17 µm^2^; *p* < 0.001) (Table 1).

Twenty-one fatty acids were identified in the sperm of Alpine and Spanish–Creole bucks. Eight of these fatty acids, namely 13:0, 16:0, 18:0, 24:0, 14:1, 18:1 (cis-9), 24:1, and 18:2, showed statistically significant differences (*p* < 0.05). The concentrations of 16:0, 18:0, 24:0, 18:1 (cis-9), and 18:2 were higher in the Alpine breed, whereas the levels of 13:0, 14:1, and 24:1 were higher in the Spanish–Creole breed (*p* < 0.05). The total fatty acid content was significantly greater in sperm from Alpine bucks (741.09 ng/mL) when compared to that of Spanish–Creole bucks (588.30 ng/mL) (*p* < 0.001). The total saturated fatty acid (SFA) content was also higher in sperm from Alpine bucks (280.78 ng/mL) as compared to that of the Spanish–Creole bucks (186.91 ng/mL) (*p* < 0.001). The amounts of cis-monounsaturated fatty acids (cis-MUFA) were 147.89 ng/mL and 169.09 ng/mL (*p* = 0.036) for the Alpine and Spanish–Creole bucks, respectively. The total polyunsaturated fatty acid (PUFA) content was 249.27 ng/mL in Alpine bucks and 184.85 ng/mL in Spanish–Creole bucks (*p* < 0.001) (Table 2).

**Figure 1:**
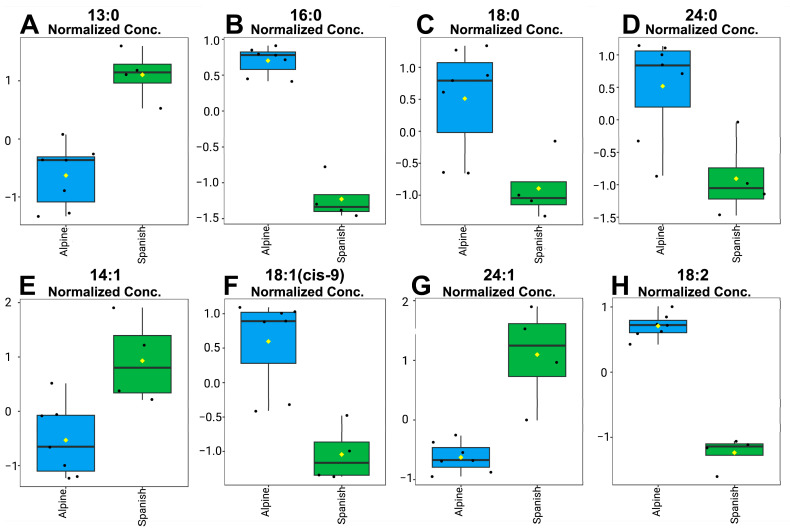
Normalized concentrations of significantly different fatty acids between Alpine and Spanish–Creole goat sperm. Each boxplot represents the normalized abundance of individual fatty acids (**A**–**H**) following cryopreservation. Data are shown for each animal (n = 7 Alpine, n = 4 Spanish–Creole), with values normalized by Z-score transformation. Statistical comparisons were performed using Welch’s *t*-test with FDR correction; only fatty acids that remained significant after FDR adjustment (*p* < 0.05) are shown. Yellow diamonds indicate group means; black dots represent individual animals.

Among saturated fatty acids (SFAs), 13:0, 16:0, 18:0, and 24:0 differed between breeds. While 13:0 was more abundant in Spanish–Creole bucks (FC = 0.547, *p* = 0.003), the levels of 16:0 (FC = 2.163, *p* < 0.001), 18:0 (FC = 1.395, *p* = 0.038), and 24:0 (FC = 1.014, *p* = 0.038) were higher in Alpine sperm. Notably, 16:0 exhibited both statistical and biological relevance. In the cis-monounsaturated fatty acid group, 14:1 (FC = 0.743, *p* = 0.033) and 24:1 (FC = 0.881, *p* = 0.003) were elevated in Spanish–Creole sperm, whereas 18:1 (cis-9) showed a marked increase in Alpine sperm (FC = 2.044, *p* = 0.007). This fatty acid was also considered biologically relevant. Linoleic acid (18:2) in the PUFA group was more abundant in Alpine bucks (FC = 2.354, *p* < 0.001), aligning with its dual statistical and biological importance. The saturation index (SI) was higher in Alpine bucks (0.61, *p* < 0.001), consistent with the overall lipidomic profile. (Table 2 and Figure 1).

**Figure 2:**
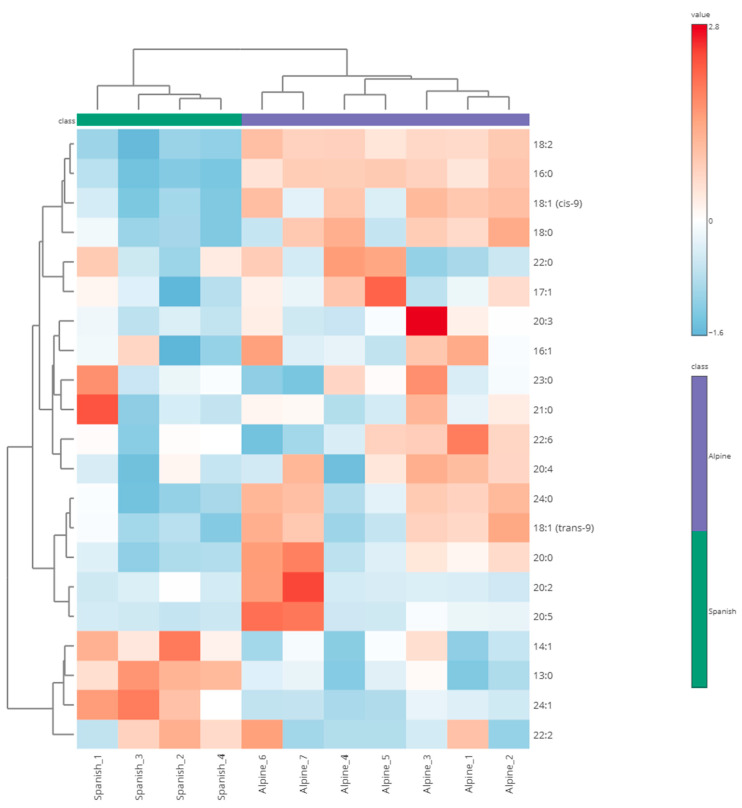
Heatmap of hierarchical clustering of differentially abundant fatty acids in sperm from Alpine and Spanish–Creole bucks. The top dendrogram depicts sample clustering by breed, while the side dendrogram clusters fatty acids based on abundance patterns. Color gradient (red: higher abundance, blue: lower abundance) indicates the relative levels of individual fatty acids.

Heatmap analysis revealed a clear clustering of fatty acid profiles between Alpine and Spanish–Creole goat sperm. Fatty acids 18:2, 16:0, and 18:1 (cis-9) were highly abundant in Alpine samples, while 24:1, 22:6, and 20:5 were more prevalent in Spanish–Creole samples. Hierarchical clustering further highlighted these differences, forming distinct groups that corresponded to each breed (Figure 2).

**Figure 3:**
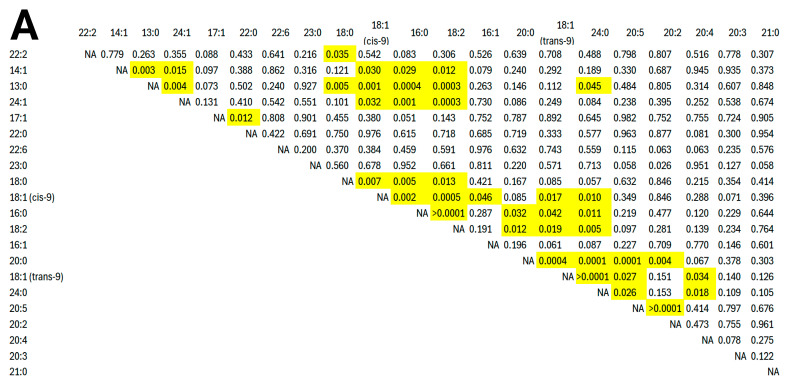

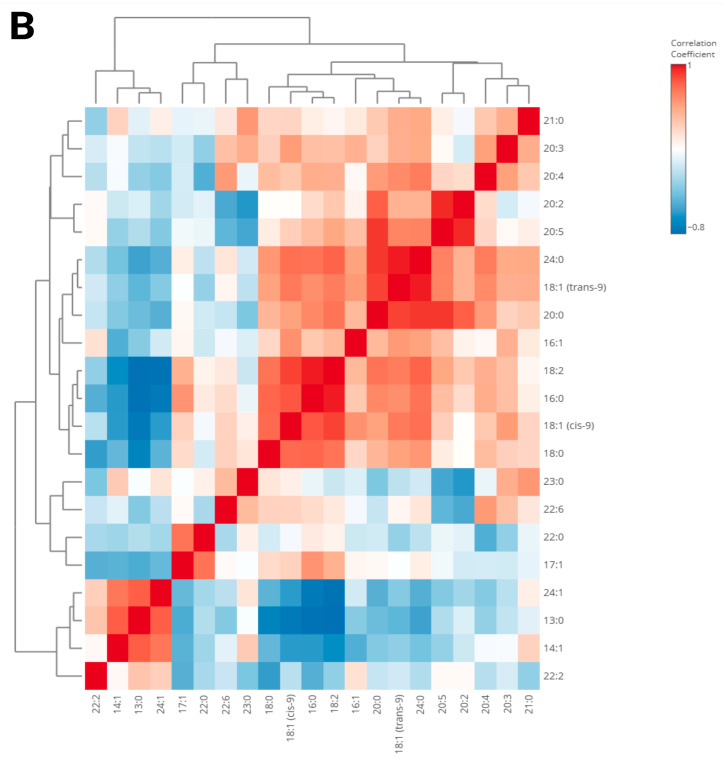
Pearson’s correlation analysis of fatty acid concentrations in sperm from individual Alpine and Spanish–Creole bucks (n = 11; pooled data). (**A**) Correlation matrix showing Pearson correlation coefficients (upper triangle) and corresponding *p*-values (lower triangle). Significant correlations (*p* < 0.05) are highlighted in yellow. (**B**) Heatmap representing Pearson’s correlation coefficients between fatty acids, with hierarchical clustering. Red and blue indicate positive and negative correlations, respectively.

Correlation analysis revealed that 22:2 exhibited a negative correlation with 18:0 (r = −0.64, *p* = 0.035). The fatty acid 14:1 was positively correlated with 13:0 (r = 0.80, *p* = 0.003) and 24:1 (r = 0.71, *p* = 0.015), whereas it showed negative correlations with 16:0 (r = −0.65, *p* = 0.029), 18:1(cis-9) (r = −0.65, *p* = 0.030), and 18:2 (r = −0.72, *p* = 0.012). The saturated fatty acid 13:0 demonstrated a positive correlation with 24:1 (r = 0.79, *p* = 0.004) and negative correlations with 18:0 (r = −0.78, *p* = 0.005), 18:1 (cis-9) (r = −0.84, *p* = 0.001), 18:2 (r = −0.89, *p* < 0.001), 24:0 (r = −0.61, *p* = 0.045), and 16:0 (r = −0.88, *p* < 0.001). Additionally, 24:1 showed negative correlations with 18:1 (cis-9) (r = −0.64, *p* = 0.032), 18:2 (r = −0.88, *p* < 0.001), and 16:0 (r = −0.67, *p* = 0.024). A positive correlation was identified between 17:1 and 22:0 (r = 0.72, *p* = 0.012). The fatty acid 23:0 was negatively correlated with 20:2 (r = −0.66, *p* = 0.026). Regarding other associations, 18:0 showed positive correlations with 18:1 (cis-9) (r = 0.75, *p* = 0.007), 16:0 (r = 0.77, *p* = 0.005), and 18:2 (r = 0.72, *p* = 0.013). In turn, 18:1 (cis-9) was positively correlated with 16:0 (r = 0.81, *p* = 0.002), 18:2 (r = 0.87, *p* < 0.001), 16:1 (r = 0.61, *p* = 0.046), 18:1 (trans-9) (r = 0.70, *p* = 0.017), and 24:0 (r = 0.73, *p* = 0.010). The fatty acid 16:0 was positively correlated with 18:2 (r = 0.96, *p* < 0.001), 20:0 (r = 0.64, *p* = 0.032), 18:1 (trans-9) (r = 0.62, *p* = 0.042), and 24:0 (r = 0.73, *p* = 0.011). Similarly, 18:2 displayed positive correlations with 20:0 (r = 0.72, *p* = 0.012), 18:1 (trans-9) (r = 0.69, *p* = 0.019), and 24:0 (r = 0.78, *p* = 0.005). Furthermore, 20:0 was positively correlated with 18:1 (trans-9) (r = 0.87, *p* < 0.001), 24:0 (r = 0.90, *p* < 0.001), 20:5 (r = 0.91, *p* < 0.001), and 20:2 (r = 0.79, *p* = 0.004). The fatty acid 18:1 (trans-9) was positively correlated with 24:0 (r = 0.96, *p* < 0.001), 20:5 (r = 0.66, *p* = 0.027), and 20:4 (r = 0.64, *p* = 0.034). In addition, 24:0 showed positive correlations with 20:5 (r = 0.66, *p* = 0.026) and 20:4 (r = 0.69, *p* = 0.018). A positive correlation was detected between 20:5 and 20:2 (r = 0.94, *p* < 0.001). A detailed summary of these relationships is presented in Figure 3.

Significant differences in fatty acid profiles were observed between Alpine and Spanish–Creole buck sperm. Fatty acid 18:2 showed the highest fold change (FC = 2.354, log2(FC) = 1.235, *p* = 1.37 × 10^−7^, −log10(p) = 6.862), followed by fatty acid 16:0 with a fold change of 2.163 (log2(FC) = 1.113, *p* = 4.15 × 10^−7^, −log10(p) = 6.382), and fatty acid 18:1 (cis-9) with a fold change of 2.044 (log2(FC) = 1.031, *p* = 1.64 × 10^−3^, −log10(p) = 2.785). These findings indicate a significant enrichment of these fatty acids in the Alpine sperm compared to Spanish–Creole sperm (Supplementary Figure S2).

**Figure 4:**
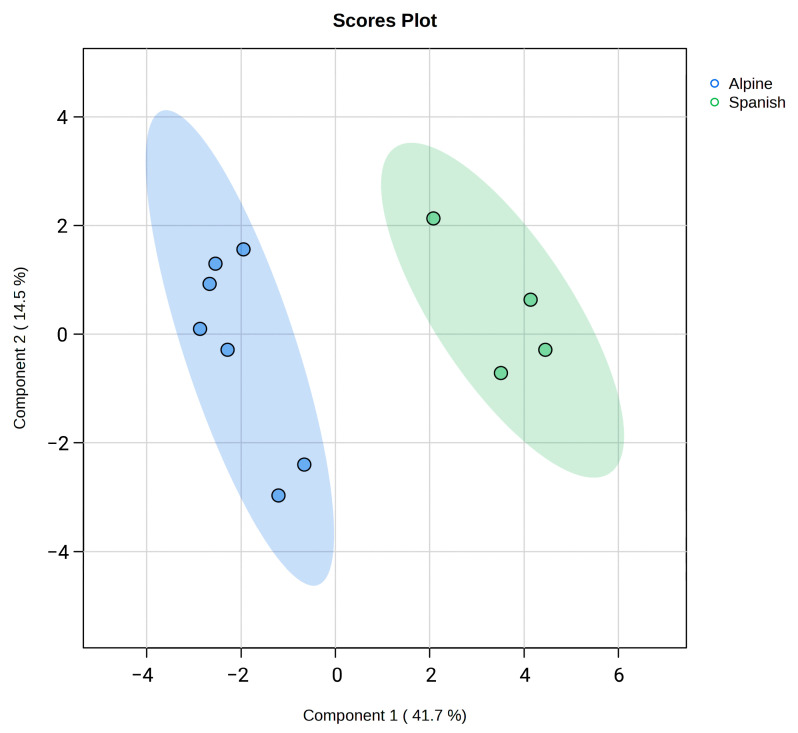
Partial Least Squares Discriminant Analysis (2D PLS-DA) of the goat sperm cell fatty acids from Alpine and Spanish–Creole goats. The plots indicate a separation between Alpine and Spanish–Creole goats.

**Figure 5:**
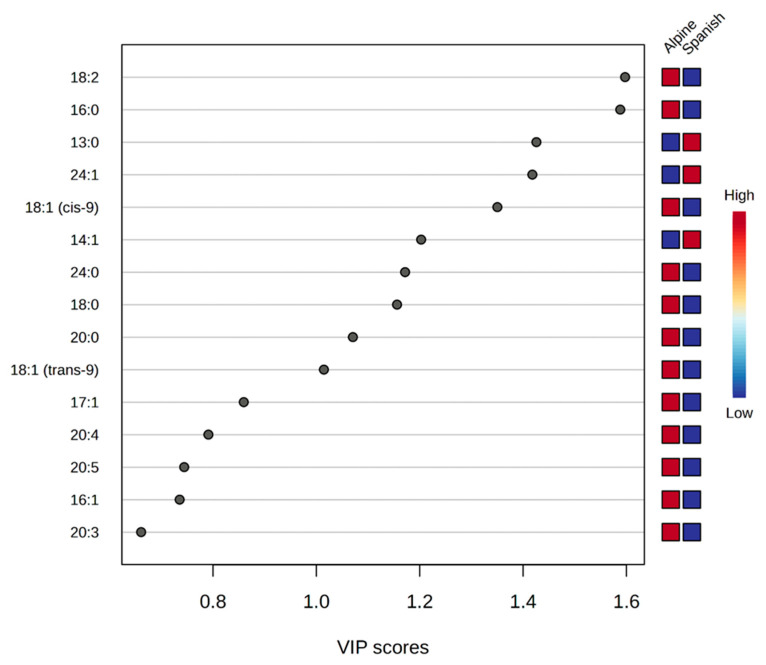
Variable importance in projection (VIP) plot displays the top 15 most important fatty acid features identified by PLS-DA. Colored boxes on the right indicate the concentration of the corresponding fatty acid from Alpine and Spanish–Creole goats. VIP score is a weighted score based on the PLS-DA model. The VIP plot highlights the most discriminative fatty acids between Alpine and Spanish–Creole breeds, with 18:2, 16:0, and 13:0 showing the highest importance scores. The concentration differences shown in the colored boxes indicate breed-specific variations in lipid composition. Specifically, the higher proportions of SFA (16:0 and 13:0) and UFA (18:2 and 24:1) may contribute to differences in membrane stability and cryotolerance between breeds. These findings highlight the role of fatty acid composition in distinguishing sperm functional characteristics in Alpine and Spanish–Creole goats.

The Principal Component Analysis (PCA) was applied to effectively distinguish Alpine and Spanish–Creole goat sperm based on their fatty acid profiles. The PC1 accounted for 42% of the total variance, clearly separating the two groups along this axis, while PC2 explained an additional 17.3% of the variance (Figure 4). Partial Least Squares Discriminant Analysis (PLS-DA) identified the key fatty acids responsible for the differences between Alpine and Spanish–Creole groups. The variable importance in projection (VIP) scores highlighted 18:2, 16:0, and 13:0 as the most significant fatty acids, with their high scores indicating their crucial roles in distinguishing the groups. Additional important contributors included 24:1 and 18:1 (cis-9), further emphasizing the distinct lipid composition of each breed (Figure 5).

**Figure 6:**
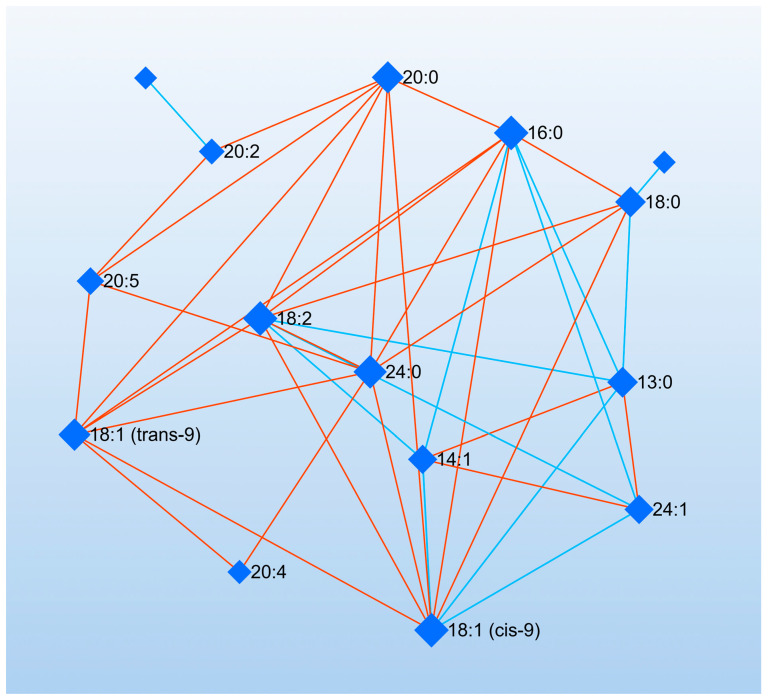
Debiased Sparse Partial Correlation (DSPC) network of fatty acids. Pathway and network analyses of the fatty acids with the highest VIP scores (16:0, 18:2, and 18:1-cis-9) and the most abundant fatty acids (20:0 and 24:0) were performed using MetScape.

Network analysis was used to identify key fatty acids with high centrality measures. Fatty acids 16:0, 18:2, and 18:1 (cis-9) had the highest degree values (value: 9), meaning they had the most connections within the network. This suggests that these fatty acids play a central role and have strong associations with other fatty acids. In terms of betweenness values, fatty acid 20:0 exhibited the highest value (20.17), followed by fatty acids 24:0 and 18:0, with values of 13.74 and 13.49, respectively. Fatty acids with lower degree and betweenness values, such as 22:2 and 23:0, were positioned more peripherally in the network and had fewer connections (Figure 6).

## 4. Discussion

The sperm lipid profiles of the two breeds exhibited distinct compositional patterns. The fatty acids that differed between sperm from Alpine and Spanish–Creole bucks included 13:0, 16:0, 18:0, 24:0, 14:1, 18:1 (cis-9), 24:1, and 18:2. Among them, 16:0, 18:1 (cis-9), and 18:2 were identified as both statistically and biologically relevant based on fold change analysis. Differences were also noted in total saturated, monounsaturated, and polyunsaturated fatty acids, as well as in total fatty acid content and saturation index. While Alpine bucks exhibited higher levels of total SFA, PUFA, and saturation index, Spanish–Creole bucks showed elevated levels of total cis-MUFA.

This study aimed to perform lipidomic profiling after cryopreservation in order to identify both breed-specific and structurally resilient membrane lipid components that withstand cryogenic stress. Although freezing and thawing are known to alter the structure and abundance of membrane lipids, previous studies have shown that interbreed differences in lipid composition remain distinguishable even after thawing [14,34]. Therefore, post-thaw lipidomic profiles can be considered as integrated indicators reflecting both inherent membrane architecture and resistance capacity to cryodamage. This approach is also practically relevant, as the ultimate goal of cryopreservation is to preserve cellular function after thawing. Hence, identifying lipid species that persist or differ significantly after thawing not only reflects biological variability but also provides valuable insights into structural membrane robustness against cryoinjury. The freeze–thaw process is known to induce structural alterations in sperm membranes [39,40,41]; however, not all lipids are equally affected. Several studies have shown that certain lipid species exhibit stability or resilience during cryopreservation and may play protective roles against cryodamage [34,42,43,44,45], while others are selectively depleted or reorganized [46,47,48]. Therefore, by analyzing post-thaw samples, this study aimed to identify persistent lipidomic signatures that remain detectable after cryostress. These enduring profiles likely represent structurally stable, breed-specific membrane traits rather than transient artifacts of the freezing process [34,45,49].

In Saanen goats, bucks with higher levels of phosphatidylcholine (PC) and triglycerides (TG) exhibited superior sperm cryotolerance [34]. The zwitterionic nature of PC stabilizes lipid bilayers and helps preserve membrane integrity by contributing to membrane charge balance and structure. In our study, SFA (280.78 ± 5.29 ng/mL) and PUFA (249.27 ± 4.53 ng/mL) were more abundant in sperm from Alpine bucks compared to Spanish–Creole bucks (SFA: 186.91 ± 8.67 ng/mL; PUFA: 184.85 ± 8.17 ng/mL; *p* < 0.001 for both), indicating breed-dependent lipidomic profiles. This aligns with previous studies suggesting breed-specific metabolic adaptations that may affect sperm membrane composition and resistance to cryodamage [28,35,50]. Sperm from Alpine bucks also exhibited elevated concentrations of palmitic acid (16:0) and linoleic acid (18:2), which are associated with enhanced membrane structure and oxidative resilience [51,52]. SFAs such as 16:0 promote tight phospholipid packing and increase membrane stability under freezing stress, thereby limiting cryo-induced damage [53,54,55]. These findings are consistent with reports indicating that higher palmitic acid levels support membrane integrity [14,50]. Additionally, stearic acid (18:0), another SFA elevated in Alpine bucks, has been associated with acrosomal stability and protection against cryo-induced capacitation [56,57]. Conversely, unsaturated fatty acids such as 18:1 (cis-9) and 18:2 introduce structural kinks that increase fluidity and support sperm functions such as capacitation and acrosomal reaction [54,58,59]. Although PUFAs are vulnerable to peroxidation, their incorporation into sperm membranes has been linked to improved post-thaw motility and membrane integrity when antioxidant defenses are sufficient [60,61,62,63]. In this study, sperm from Spanish–Creole bucks showed higher levels of total cis-monounsaturated fatty acids (cis-MUFA), particularly 18:1 (cis-9), which are known to enhance membrane flexibility and to facilitate sperm–oocyte fusion and zona binding [14,28,64]. Membrane fluidity is not only vital for sperm motility but also plays a critical role during fertilization, including oviductal transport and penetration of the zona pellucida. These fatty acids may also influence intracellular signaling pathways associated with sperm capacitation and acrosome reaction. As such, breed-specific enrichment of particular lipid classes might reflect long-term physiological adaptations to reproductive demands under diverse environmental conditions. The PUFA profile has been identified as a key factor underlying membrane dynamics, supporting the idea that the higher PUFA content observed in Alpine bucks may represent an adaptive advantage [35]. Moreover, balanced proportions of SFA and PUFA have been suggested to contribute synergistically to maintaining both membrane rigidity and fluidity, which are essential for cryotolerance [28,56].

Linoleic acid (18:2), a polyunsaturated fatty acid (PUFA), has been reported to enhance membrane fluidity and structural flexibility by introducing double bonds that disrupt the tight packing of phospholipid bilayers, thereby increasing lateral mobility of membrane components and improving functional outcomes such as the acrosome reaction and fertilization capacity [55,58,65,66,67]. Its incorporation into sperm membranes has been associated with improved resistance to cold-induced phase transitions during cryopreservation, which often results in structural damage [65,66,67]. Experimental studies in sperm from rams and buffaloes support the role of linoleic acid supplementation in improving post-thaw sperm motility and membrane integrity while reducing structural and morphological abnormalities [68,69]. These beneficial effects are linked to increased osmotic resistance and reduced lipid peroxidation, both of which are vital for preserving sperm viability post-thaw [70,71]. Additionally, 18:2 abundance has been correlated with improved motility and fertilization potential in various species [14,58]. In our study, the elevated levels of 18:2, 16:0, and 18:0 in Alpine bucks reflect a lipidomic profile associated with higher cryotolerance. The coordinated elevation of these fatty acids may represent an inherent trait contributing to improved membrane stability during the freeze–thaw process. These findings are consistent with previous studies in bovine and human sperm, which linked higher SFA and PUFA proportions to improved post-thaw viability and motility [56,64]. The breed-specific differences in lipid profiles suggest a genetically influenced balance between membrane rigidity and fluidity that could influence cryosurvival, as sperm with higher 16:0, 18:2, and total PUFA levels favor plasma membrane stabilization during cryopreservation [14,28,50]. Such compositional traits may act synergistically with species-specific cryoprotective mechanisms to sustain sperm functionality. Moreover, the interplay between dietary lipid sources, genetic selection, and membrane remodeling during spermatogenesis could ultimately determine sperm freezability. High PUFA levels have been correlated with enhanced motility and viability in human sperm [72], and elevated 18:2 concentrations may contribute to improved sperm motility by increasing membrane fluidity [73]. Further evidence suggests that PUFA-mediated enhancement of membrane dynamics is critical for cold tolerance [35]. These results reinforce the potential value of targeting specific lipid classes to optimize reproductive biotechnologies across breeds. Future investigations integrating molecular lipid markers with functional sperm analyses may further clarify the mechanistic pathways underlying cryotolerance.

The differential abundance of saturated (13:0, 16:0, 18:0, and 24:0) and unsaturated fatty acids (18:1 cis-9, 24:1, and 18:2) across breeds suggests breed-specific regulatory mechanisms influencing lipid metabolism, potentially mediated by transcriptional regulators such as Sterol Regulatory Element-Binding Proteins (SREBPs) and Acyl-CoA Synthetase Long-Chain Family Members (ACSLs) [74,75]. These lipids play essential roles in stabilizing phospholipid bilayers and enhancing membrane flexibility under freezing conditions [64,76,77]. Additionally, environmental factors such as nutrition, seasonal variation, and thermal stress may further modulate the balance between saturated and unsaturated fatty acids, affecting sperm motility, membrane stability, and overall cryotolerance [78]. It is important to clarify that the breeds examined were not selected for known cryopreservation performance but represent regionally relevant genotypes maintained under standardized management. Therefore, the observed lipidomic differences likely reflect intrinsic breed-specific traits rather than the result of artificial selection for cryoresistance.

PCA indicated lipidomic differences between Alpine and Spanish–Creole buck sperm. VIP scores identified 18:2, 16:0, 13:0, 24:1, and 18:1 (cis-9) as the most influential contributors to this separation. Given its role in arachidonic acid biosynthesis, 18:2 may be involved in reproductive signaling pathways. Elevated levels of 16:0 and 13:0 could suggest enhanced membrane rigidity, potentially contributing to structural resilience under cryogenic stress. Differences in 24:1 and 18:1 (cis-9) may reflect alterations in membrane flexibility and fusion capacity. These variations might be associated with metabolic processes such as elongation (carbon chain extension) and desaturation (double bond formation), indicating a breed-specific lipid remodeling that could influence sperm membrane organization and function.

Our findings demonstrate distinct breed-associated differences in the sperm lipidome of goats, suggesting that specific lipid species may be under the influence of genetic polymorphisms linked to male reproductive function [61,79]. Further research on environmental factors and nutrition might yield relevant information [80]. As such, future research on environmental exposures, food, and genetic changes can improve metabolic features, such as lipid profiles, in livestock productivity and reproduction.

## 5. Conclusions

This study provides a comparative lipidomic analysis of sperm from Alpine and Spanish–Creole goat breeds, revealing significant breed-specific differences in lipid composition. The fatty acids 16:0, 18:1 (cis-9), and 18:2 were statistically and physiologically significant, underscoring their roles in maintaining structural integrity, membrane fluidity, and overall sperm functionality. These findings emphasize the critical interrelationships between lipid metabolism and sperm cryotolerance. Incorporating lipidomic profiling into the development of advanced cryopreservation techniques and breeding soundness exams could enhance the post-thaw sperm’s viability, offering valuable benefits for artificial insemination programs and genetic preservation projects.

## Figures and Tables

**Table 1:** The post-thaw values for motility and kinematic parameters of Alpine and Spanish–Creole goat breeds.

Parameters	Alpine Mean ± SEM	Spanish–Creole Mean ± SEM	*p*-Values
Total Motility (%)	60.28 ± 0.81	60.82 ± 0.97	0.67
Progressive Motility (%)	36.3 ± 2.03	40.88 ± 1.65	0.109
VAP (µm/s)	104.68 ± 6.38	100.28 ± 6.13	0.633
VCL (µm/s)	214.34 ± 13.15	197.61 ± 13.99	0.398
VSL (µm/s)	79.28 ± 5.09	72.51 ± 3.6	0.318
ALH (µm)	10.01 ± 0.48	9.53 ± 0.62	0.537
BCF (Hz) *	33.29 ± 0.88	29.67 ± 1.11	0.017
LIN (%)	40.66 ± 1.54	39.86 ± 1.35	0.709
STR (%)	76.08 ± 1.3	74.34 ± 1.81	0.432
AREA (µm^2^) *	17.65 ± 0.11	18.48 ± 0.17	<0.001
WOB (%)	52.01 ± 1.05	52.57 ± 0.72	0.683
ELONGATION	0.48 ± 0.01	0.48 ± 0.01	0.886

* Indicates that it has statistical differences between Alpine and Spanish–Creole breeds (*p* < 0.05).

**Table 2:** Average fatty acid concentration (ng/200 × 10^6^ cells) between Alpine and Spanish–Creole goats. Saturation index = SFA/UFA.

Fatty Acid	Alpine Mean (ng/mL) ± SEM	Spanish–Creole Mean (ng/mL) ± SEM	Fold Change (FC)	−log10 (*p*-Value)	FDR-Corrected *p*-Value
13:0 *	11.23 ± 1.10	20.54 ± 1.19	0.547	3.369	0.003
16:0 *^+^	166.21 ± 3.42	76.84 ± 7.13	2.163	6.382	<0.001
18:0 *	46.52 ± 2.96	33.36 ± 2.40	1.395	1.837	0.038
20:0	2.06 ± 0.12	1.61 ± 0.06	1.283	1.551	0.066
21:0	9.59 ± 0.02	9.59 ± 0.08	1.000	0.014	0.968
22:0	10.62 ± 0.02	10.61 ± 0.02	1.001	0.111	0.814
23:0	16.51 ± 0.08	16.57 ± 0.10	0.996	0.184	0.724
24:0 *	18.04 ± 0.05	17.80 ± 0.05	1.014	1.896	0.038
Total SFA *	280.78 ± 5.29	186.91 ± 8.67	1.502	5.395	<0.001
14:1 *	86.45 ± 5.18	116.36 ± 8.06	0.743	2.020	0.033
16:1	11.63 ± 1.33	8.09 ± 2.04	1.437	0.786	0.245
17:1	14.37 ± 0.85	11.90 ± 0.91	1.207	1.020	0.182
18:1 (cis-9) *^+^	11.57 ± 0.90	5.66 ± 0.74	2.044	2.785	0.007
24:1 *	23.86 ± 0.18	27.07 ± 0.77	0.881	3.298	0.003
Total cis-MUFA *	147.89 ± 4.69	169.09 ± 8.02	0.875	1.445	0.036
18:1 (trans-9)	63.14 ± 4.52	47.45 ± 3.33	1.331	1.389	0.086
Total trans-MUFA	63.14 ± 4.52	47.45 ± 3.33	1.331	1.389	0.086
18:2 *^+^	94.45 ± 2.01	40.12 ± 3.47	2.354	6.862	<0.001
20:2	18.48 ± 0.60	17.71 ± 0.16	1.044	0.430	0.459
20:3	13.17 ± 0.42	12.42 ± 0.12	1.061	0.666	0.302
20:4	20.88 ± 0.53	19.56 ± 0.46	1.067	0.885	0.228
20:5	18.74 ± 0.68	17.31 ± 0.05	1.082	0.801	0.245
22:2	9.33 ± 0.11	9.53 ± 0.11	0.979	0.582	0.344
22:6	74.23 ± 5.90	68.21 ± 4.24	1.088	0.301	0.583
Total PUFA *	249.27 ± 4.53	184.85 ± 8.17	1.349	4.459	<0.001
Total Fatty Acid *	741.09 ± 11.43	588.30 ± 23.17	1.260	4.050	<0.001
Saturation Index *	0.61 ± 0.02	0.47 ± 0.01	1.310	3.508	<0.001

* Indicates that it has statistical differences between Alpine and Spanish–Creole breeds (*p* < 0.05). ^+^ Indicates that it has statistical and biological differences between Alpine and Spanish–Creole breeds (*p* < 0.05, FC > 2).

## Data Availability

The data presented in this study are available upon request from the corresponding author due to privacy.

## Supplementary Materials

The following supporting information can be downloaded at: https://www.mdpi.com/article/10.3390/ani15131897/s1, Figure S1: Calibration curve of internal and external standards; Figure S2: Volcano plot displaying significant differences in fatty acid profiles were development between Alpine and Spanish-Creole goat sperm.

## References

[1] Bodu M., Hitit M., Memili E. (2025). Harnessing the value of fertility biomarkers in bull sperm for buck sperm. Anim. Reprod. Sci..

[2] Alemayehu G., Mamo G., Alemu B., Desta H., Wieland B. (2021). Towards objective measurement of reproductive performance of traditionally managed goat flocks in the drylands of Ethiopia. Trop. Anim. Health Prod..

[3] Delgadillo J.A., Martin G.B. (2015). Alternative methods for control of reproduction in small ruminants: A focus on the needs of grazing industries. Anim. Front..

[4] Nissen H.P., Kreysel H.W. (1983). Analysis of phospholipids in human semen by high-performance liquid chromatography. J. Chromatogr. B Biomed. Sci. Appl..

[5] Lenzi A., Picardo M., Gandini L., Dondero F. (1996). Lipids of the sperm plasma membrane: From polyunsaturated fatty acids considered as markers of sperm function to possible scavenger therapy. Hum. Reprod. Update.

[6] Bodu M., Hitit M., Greenwood O.C., Murray R.D., Memili E. (2025). Extender development for optimal cryopreservation of buck sperm to increase reproductive efficiency of goats. Front. Vet. Sci..

[7] Lucio C., Brito M., Angrimani D., Belaz K., Morais D., Zampieri D., Losano J., Assumpção M., Nichi M., Eberlin M. (2017). Lipid composition of the canine sperm plasma membrane as markers of sperm motility. Reprod. Domest. Anim..

[8] van der Horst G., Maree L. (2022). Origin, Migration, and Reproduction of Indigenous Domestic Animals with Special Reference to Their Sperm Quality. Animals.

[9] Pan X., Kang G., Yang N., Zhang X., Shao L., Zhai P., Feng Q., Zhang X., Li J., Wang X. (2022). A Sperm Quality Detection System Based on Microfluidic Chip and Micro-Imaging System. Front. Vet. Sci..

[10] Hitit M., Özbek M., Ayaz-Guner S., Guner H., Oztug M., Bodu M., Kirbas M., Bulbul B., Bucak M.N., Ataman M.B. (2021). Proteomic fertility markers in ram sperm. Anim. Reprod. Sci..

[11] Kameni S.L., Dlamini N.H., Feugang J.M. (2024). Exploring the Full Potential of Sperm Function With Nanotechnology Tools. Anim. Reprod..

[12] Sigit B., Panjono, Riyan Nugroho A. The Effect of Sanrego Wood (Lunasia amara Blanco) Extract Addition to the Andromed^®^ Diluent on Sperm Quality of Belgian Blue Crossbreeds Bull. Proceedings of the 2nd International Conference on Smart and Innovative Agriculture (ICoSIA 2021).

[13] Zhang H., Liu H., Kataoka S., Kinukawa M., Uchiyama K., Kambe J., Watanabe G., Jin W., Nagaoka K. (2021). L-amino acid oxidase 1 in sperm is associated with reproductive performance in male mice and bulls. Biol. Reprod..

[14] Evans H.C., Dinh T.T.N., Ugur M.R., Hitit M., Sajeev D., Kaya A., Topper E., Nicodemus M.C., Smith G.D., Memili E. (2020). Lipidomic markers of sperm cryotolerance in cattle. Sci. Rep..

[15] Lopes César M., Maicon Pereira L., Emmanuel Emydio Gomes P., Rafael Alexandre M., Shumaila M., Muhammad A. (2023). Alternative Animal Feeding for Intensive Livestock Farming Systems and Their Impact on Reproductive Performance of Ruminants. Intensive Animal Farming.

[16] Congras A., Yerle-Bouissou M., Pinton A., Vignoles F., Liaubet L., Ferchaud S., Acloque H. (2014). Sperm DNA Methylation Analysis in Swine Reveals Conserved and Species-Specific Methylation Patterns and Highlights an Altered Methylation at the GNAS Locus in Infertile Boars1. Biol. Reprod..

[17] Namani S.C., Kolikapongu R.S., Chelkapally S.C., Heikal R., Neha A., Shaik A., Pech Cervantes A.A., Whitley N.C., Kouakou B., Terrill T.H. (2023). 379 Impacts of Serecia Lespedeza and Black Seed Meal Dietary Supplementation on Sperm Quality and Fertilityvariables of Male Goats. J. Anim. Sci..

[18] Safarinejad M.R., Hosseini S.Y., Dadkhah F., Asgari M.A. (2010). Relationship of omega-3 and omega-6 fatty acids with semen characteristics, and anti-oxidant status of seminal plasma: A comparison between fertile and infertile men. Clin. Nutr..

[19] Furimsky A., Vuong N., Xu H., Kumarathasan P., Xu M., Weerachatyanukul W., Bou Khalil M., Kates M., Tanphaichitr N. (2005). Percoll Gradient-Centrifuged Capacitated Mouse Sperm Have Increased Fertilizing Ability and Higher Contents of Sulfogalactosylglycerolipid and Docosahexaenoic Acid-Containing Phosphatidylcholine Compared to Washed Capacitated Mouse Sperm1. Biol. Reprod..

[20] Sellem E., Jammes H., Schibler L. (2022). Sperm-borne sncRNAs: Potential biomarkers for semen fertility?. Reprod. Fertil. Dev..

[21] Evans H.C., Dinh T.T.N., Hardcastle M.L., Gilmore A.A., Ugur M.R., Hitit M., Jousan F.D., Nicodemus M.C., Memili E. (2021). Advancing Semen Evaluation Using Lipidomics. Front. Vet. Sci..

[22] Ding N., Zhang Y., Wang J., Liu J., Zhang J., Zhang C., Zhou L., Cao J., Jiang L. (2025). Lipidomic and transcriptomic characteristics of boar seminal plasma extracellular vesicles associated with sperm motility. Biochim. Biophys. Acta (BBA)-Mol. Cell Biol. Lipids.

[23] Prasinou P., De Amicis I., Fusaro I., Bucci R., Cavallini D., Parrillo S., Caputo M., Gramenzi A., Carluccio A. (2023). The Lipidomics of Spermatozoa and Red Blood Cells Membrane Profile of Martina Franca Donkey: Preliminary Evaluation. Animals.

[24] Cheng X., Xie H., Xiong Y., Sun P., Xue Y., Li K. (2023). Lipidomics profiles of human spermatozoa: Insights into capacitation and acrosome reaction using UPLC-MS-based approach. Front. Endocrinol..

[25] Furse S., Kusinski L.C., Ray A., Glenn-Sansum C., Williams H.E.L., Koulman A., Meek C.L. (2022). Relative Abundance of Lipid Metabolites in Spermatozoa across Three Compartments. Int. J. Mol. Sci..

[26] Singh A., Singh V., Narwade B., Mohanty T., Atreja S. (2012). Comparative Quality Assessment of Buffalo (*Bubalus bubalis*) Semen Chilled (5 °C) in Egg Yolk- and Soya Milk–Based Extenders. Reprod. Domest. Anim..

[27] Macías García B., González Fernández L., Ortega Ferrusola C., Morillo Rodríguez A., Gallardo Bolaños J.M., Rodríguez Martinez H., Tapia J.A., Morcuende D., Peña F.J. (2011). Fatty acids and plasmalogens of the phospholipids of the sperm membranes and their relation with the post-thaw quality of stallion spermatozoa. Theriogenology.

[28] Mandal R., Badyakar D., Chakrabarty J. (2014). Role of Membrane Lipid Fatty Acids in Sperm Cryopreservation. Adv. Androl..

[29] Zadeh Hashem E., Haddad R., Eslami M. (2017). Evaluation of ram semen enrichment with oleic acid on different spermatozoa parameters during low temperature liquid storage. Small Rumin. Res..

[30] Hashem N.M., Gonzalez-Bulnes A. (2021). Nanotechnology and Reproductive Management of Farm Animals: Challenges and Advances. Animals.

[31] Bucak M.N., Karaşör Ö.F., Sarı A., Bodu M., Ili P., Narlıçay S., Ataman M.B., Sari F. (2024). Lipid mixtures (from a liposome kit) and melatonin improve post-thawed Angora goat sperm parameters. Cryobiology.

[32] Li C., Liang J., Allai L., Badaoui B., Shao Q., Ouyang Y., Wu G., Quan G., Lv C. (2024). Integrating proteomics and metabolomics to evaluate impact of semen collection techniques on the quality and cryotolerance of goat semen. Sci. Rep..

[33] Öztürk A.E., Bodu M., Bucak M.N., Ağır V., Özcan A., Keskin N., İli P., Topraggaleh T.R., Sidal H., Başpınar N. (2020). The synergistic effect of trehalose and low concentrations of cryoprotectants can improve post-thaw ram sperm parameters. Cryobiology.

[34] Xu B., Wang R., Wang Z., Liu H., Wang Z., Zhang W., Zhang Y., Su R., Liu Z., Liu Y. (2022). Evaluation of lipidomic change in goat sperm after cryopreservation. Front. Vet. Sci..

[35] Wood P.L., Scoggin K., Ball B.A., Troedsson M.H., Squires E.L. (2016). Lipidomics of equine sperm and seminal plasma: Identification of amphiphilic (O-acyl)-ω-hydroxy-fatty acids. Theriogenology.

[36] Araujo P., Nguyen T.-T., Frøyland L., Wang J., Kang J.X. (2008). Evaluation of a rapid method for the quantitative analysis of fatty acids in various matrices. J. Chromatogr. A.

[37] Xia J., Sinelnikov I.V., Han B., Wishart D.S. (2015). MetaboAnalyst 3.0—Making metabolomics more meaningful. Nucleic Acids Res..

[38] Basu S., Duren W., Evans C.R., Burant C.F., Michailidis G., Karnovsky A. (2017). Sparse network modeling and metscape-based visualization methods for the analysis of large-scale metabolomics data. Bioinformatics.

[39] Watson P.F. (2000). The causes of reduced fertility with cryopreserved semen. Anim. Reprod. Sci..

[40] Purdy P. (2006). A review on goat sperm cryopreservation. Small Rumin. Res..

[41] Yeste M. (2016). Sperm cryopreservation update: Cryodamage, markers, and factors affecting the sperm freezability in pigs. Theriogenology.

[42] Hu J.H., Zhao X.L., Tian W.Q., Zan L.S., Li Q.W. (2011). Effects of vitamin E supplementation in the extender on frozen-thawed bovine semen preservation. Animal.

[43] Zhang W., Li Y., Zhu Z. (2022). Carboxylated ε-Poly-L-Lysine Supplementation of the Freezing Extender Improves the Post-Thawing Boar Sperm Quality. Animals.

[44] Zhang W., Min L., Li Y., Lang Y., Hoque S.A.M., Adetunji A.O., Zhu Z. (2022). Beneficial Effect of Proline Supplementation on Goat Spermatozoa Quality during Cryopreservation. Animals.

[45] Zhang Y., Yuan W., Liu Y., Liu Y., Liang H., Xu Q., Liu Z., Weng X. (2023). Plasma membrane lipid composition and metabolomics analysis of Yorkshire boar sperms with high and low resistance to cryopreservation. Theriogenology.

[46] Lee S.-H., Kim Y.-J., Ho Kang B., Park C.-K. (2019). Effect of nicotinic acid on the plasma membrane function and polyunsaturated fatty acids composition during cryopreservation in boar sperm. Reprod. Domest. Anim..

[47] Lee W.H., Kim W.H., Cheong H.T., Yang B.K., Park C.K. (2019). Effect of Alpha-Linolenic Acid with Bovine Serum Albumin or Methyl-Beta-Cyclodextrin on Membrane Integrity and Oxidative Stress of Frozen-Thawed Boar Sperm. Dev. Reprod..

[48] Carro M.D.L.M., Ramírez-Vasquez R.R.A., Peñalva D.A., Buschiazzo J., Hozbor F.A. (2021). Desmosterol Incorporation Into Ram Sperm Membrane Before Cryopreservation Improves in vitro and in vivo Fertility. Front. Cell Dev. Biol..

[49] Carro M., Luquez J.M., Peñalva D.A., Buschiazzo J., Hozbor F.A., Furland N.E. (2022). PUFA-rich phospholipid classes and subclasses of ram spermatozoa are unevenly affected by cryopreservation with a soybean lecithin-based extender. Theriogenology.

[50] Macías García B., González Fernández L., Ortega Ferrusola C., Salazar-Sandoval C., Morillo Rodríguez A., Rodríguez Martinez H., Tapia J., Morcuende D., Peña F. (2011). Membrane Lipids of the Stallion Spermatozoon in Relation to Sperm Quality and Susceptibility to Lipid Peroxidation. Reprod. Domest. Anim..

[51] Zhang Y., Ding N., Cao J., Zhang J., Liu J., Zhang C., Jiang L. (2024). Proteomics and Metabolic Characteristics of Boar Seminal Plasma Extracellular Vesicles Reveal Biomarker Candidates Related to Sperm Motility. J. Proteome Res..

[52] Islam M.M., Umehara T., Tsujita N., Shimada M. (2021). Saturated fatty acids accelerate linear motility through mitochondrial ATP production in bull sperm. Reprod. Med. Biol..

[53] Klaiwattana P., Srisook K., Srisook E., Vuthiphandchai V., Neumvonk J. (2016). Effect of cryopreservation on lipid composition and antioxidant enzyme activity of seabass (*Lates calcarifer*) sperm. Iran. J. Fish. Sci..

[54] Chakrabarty J., Banerjee D., Pal D., De J., Ghosh A., Majumder G.C. (2007). Shedding off specific lipid constituents from sperm cell membrane during cryopreservation. Cryobiology.

[55] Paventi G., Di Iorio M., Rusco G., Sobolev A.P., Cerolini S., Antenucci E., Spano M., Mannina L., Iaffaldano N. (2022). The Effect of Semen Cryopreservation Process on Metabolomic Profiles of Turkey Sperm as Assessed by NMR Analysis. Biology.

[56] Kogan T., Grossman Dahan D., Laor R., Argov-Argaman N., Zeron Y., Komsky-Elbaz A., Kalo D., Roth Z. (2021). Association between Fatty Acid Composition, Cryotolerance and Fertility Competence of Progressively Motile Bovine Spermatozoa. Animals.

[57] Cerolini S., Maldjian A., Surai P., Noble R. (2000). Viability, susceptibility to peroxidation and fatty acid composition of boar semen during liquid storage1The present trial is part of an International Patent Application n. PCT/GB97/01735.1. Anim. Reprod. Sci..

[58] Lee S.-H., Kim Y.-J., Kang B.H., Yun Y.S., Park C.-K. (2020). The relationship between acrosome reaction and polyunsaturated fatty acid composition in boar sperm. Reprod. Domest. Anim..

[59] Beirão J., Zilli L., Vilella S., Cabrita E., Schiavone R., Herráez M.P. (2012). Improving Sperm Cryopreservation with Antifreeze Proteins: Effect on Gilthead Seabream (*Sparus aurata*) Plasma Membrane Lipids1. Biol. Reprod..

[60] Akbarzadeh-Jahromi M., Jafari F., Parsanezhad M.E., Alaee S. (2022). Evaluation of supplementation of cryopreservation medium with gallic acid as an antioxidant in quality of post-thaw human spermatozoa. Andrologia.

[61] Li C., Lv C., Larbi A., Liang J., Yang Q., Wu G., Quan G. (2024). Revisiting the Injury Mechanism of Goat Sperm Caused by the Cryopreservation Process from a Perspective of Sperm Metabolite Profiles. Int. J. Mol. Sci..

[62] Zhang B., Wang Y., Wu C., Qiu S., Chen X., Cai B., Xie H. (2021). Freeze-thawing impairs the motility, plasma membrane integrity and mitochondria function of boar spermatozoa through generating excessive ROS. BMC Vet. Res..

[63] Maldjian A., Pizzi F., Gliozzi T., Cerolini S., Penny P., Noble R. (2005). Changes in sperm quality and lipid composition during cryopreservation of boar semen. Theriogenology.

[64] Martínez-Soto J.C., Landeras J., Gadea J. (2013). Spermatozoa and seminal plasma fatty acids as predictors of cryopreservation success. Andrology.

[65] Ofosu J., Nartey M.A., Mo X., Ye J., Zhang Y., Zeng C., Zhang M., Fang Y., Zhou G. (2023). Ram sperm cryopreservation disrupts metabolism of unsaturated fatty acids. Theriogenology.

[66] Leão B.C.S., Rocha-Frigoni N.A.S., Cabral E.C., Coelho M.B., Ferreira C.R., Eberlin M.N., Accorsi M.F., Nogueira É., Mingoti G.Z. (2015). Improved embryonic cryosurvival observed after in vitro supplementation with conjugated linoleic acid is related to changes in the membrane lipid profile. Theriogenology.

[67] Melville D.F., Johnston S.D., Miller R.R. (2012). Flying-fox (*Pteropus* spp.) sperm membrane fatty acid composition, its relationship to cold shock injury and implications for cryopreservation success. Cryobiology.

[68] Elsherbiny H., Ghallab A., Abou-Ahmed M. (2024). Influence of linoleic acid supplementation on the quality of frozen-thawed ram semen. Vet. Med. J..

[69] Büyükleblebici S., Taşdemir U., Tuncer P.B., Durmaz E., Özgürtaş T., Büyükleblebici O., Coşkun E., Gürcan İ.S. (2014). Can linoleic acid improve the quality of frozen thawed bull sperm?. Cryoletters.

[70] Díaz R., Quiñones J., Short S., Contreras P., Ulloa-Rodríguez P., Cancino-Baier D., Sepúlveda N., Valdebenito I., Farías J.G. (2021). Effect of exogenous lipids on cryotolerance of Atlantic salmon (*Salmo salar*) spermatozoa. Cryobiology.

[71] Soares M.P., Brandelli A., Celeghini E.C.C., de Arruda R.P., Rodriguez S.A.F. (2013). Effect of cis-9,trans-11 and trans-10,cis-12 isomers of conjugated linoleic acid on the integrity and functionality of cryopreserved bovine spermatozoa. Cryobiology.

[72] Andersen J.M., Rønning P.O., Herning H., Bekken S.D., Haugen T.B., Witczak O. (2016). Fatty acid composition of spermatozoa is associated with BMI and with semen quality. Andrology.

[73] Güngör İ.H., Koca R.H., Cinkara S.D., Acısu T.C., Erişir F.E., Arkalı G., Kaya Ş.Ö., Sönmez M., Gür S., Yılmaz Ö. (2025). Changes in fatty acids, vitamins, cholesterol and amino acid profiles of ram semen by freeze-thawing process. Reprod. Biol..

[74] Shan S., Xu F., Hirschfeld M., Brenig B. (2021). Sperm Lipid Markers of Male Fertility in Mammals. Int. J. Mol. Sci..

[75] Argov-Argaman N., Mahgrefthe K., Zeron Y., Roth Z. (2013). Season-induced variation in lipid composition is associated with semen quality in Holstein bulls. Reproduction.

[76] Zhu Z., Li R., Feng C., Liu R., Zheng Y., Hoque S.A.M., Wu D., Lu H., Zhang T., Zeng W. (2020). Exogenous Oleic Acid and Palmitic Acid Improve Boar Sperm Motility via Enhancing Mitochondrial Β-Oxidation for ATP Generation. Animals.

[77] Benko F., Árvay J., Jančo I., Ďuračka M., Mohammadi-Sangcheshmeh A., Lukáč N., Ivanič P., Tvrdá E. (2024). In vitro versus cryo-induced capacitation of bovine spermatozoa, part 3: Compositional and molecular changes to the plasma membrane. Cryobiology.

[78] Gautier C., Aurich C. (2022). “Fine feathers make fine birds”—The mammalian sperm plasma membrane lipid composition and effects on assisted reproduction. Anim. Reprod. Sci..

[79] Zang S., Zou S., Chen X., Pan B., Ning A., Qin J., Wei Y., Du K., Ye J., Liang Q. (2024). Abnormalities in mitochondrial energy metabolism induced by cryopreservation negatively affect goat sperm motility. Front. Vet. Sci..

[80] Setiawan R., Christi R.F., Alhuur K.R.G., Widyastuti R., Solihati N., Rasad S.D., Hidajat K., Do D.N. (2024). Impact of glucose and pyruvate on adenosine triphosphate production and sperm motility in goats. Anim. Biosci..

