# Supplementary material for: Lipidomic Landscapes of Cryopreserved Sperm from Alpine and Spanish–Creole Bucks
Source: Animals (Basel). 2025 Jun 27;15(13):1897. doi: 10.3390/ani15131897 (PMC12248898; doi:10.3390/ani15131897)
Supplement: Supplementary file 1 [file animals-15-01897-s001.zip › Supplementary Figure S1.pdf]

Figure Legends

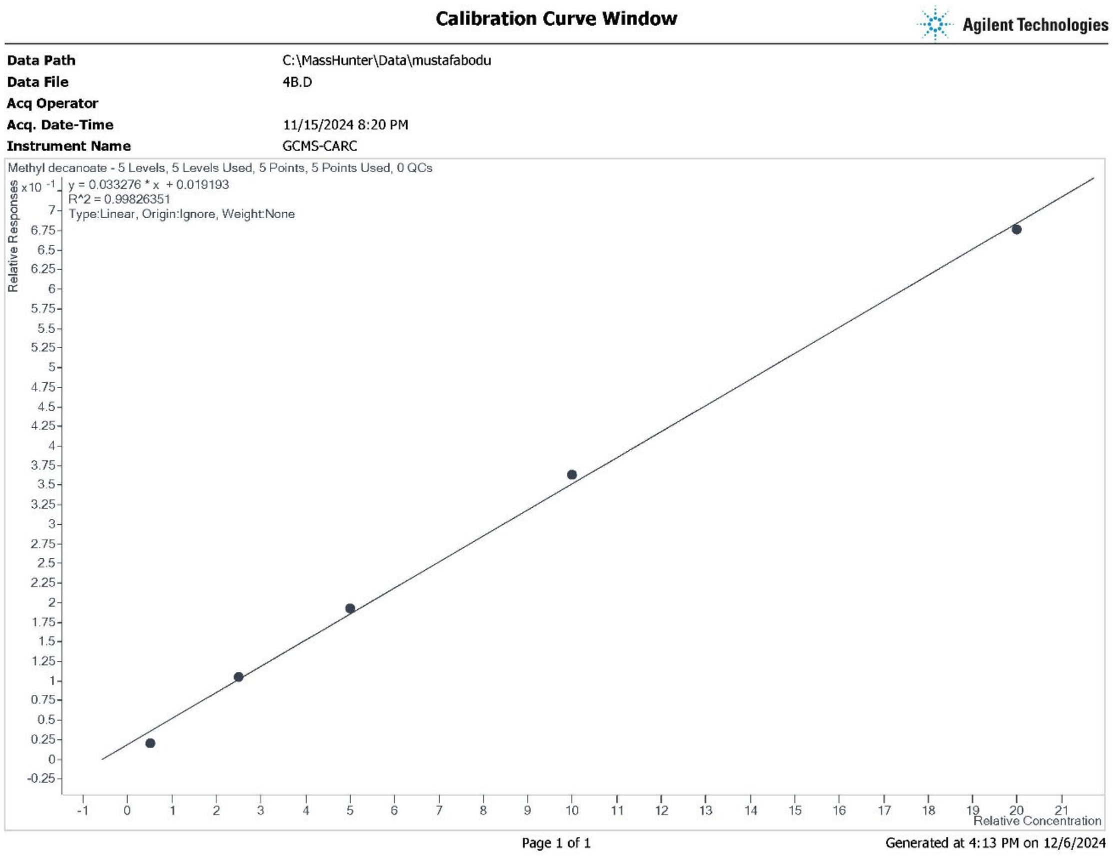

Figure S1. Calibration curve of internal and external standards. Dilutions were then made using 0, 10, 50, 100, 200, and 400 µg/ml of external standards, and each spiked with 20 µg of the internal standard, and a calibration curve was created.
