# Supplementary material for: Lipidomic Landscapes of Cryopreserved Sperm from Alpine and Spanish–Creole Bucks
Source: Animals (Basel). 2025 Jun 27;15(13):1897. doi: 10.3390/ani15131897 (PMC12248898; doi:10.3390/ani15131897)
Supplement: Supplementary file 1 [file animals-15-01897-s001.zip › Supplementary Figure S2.pdf]

Figure Legends

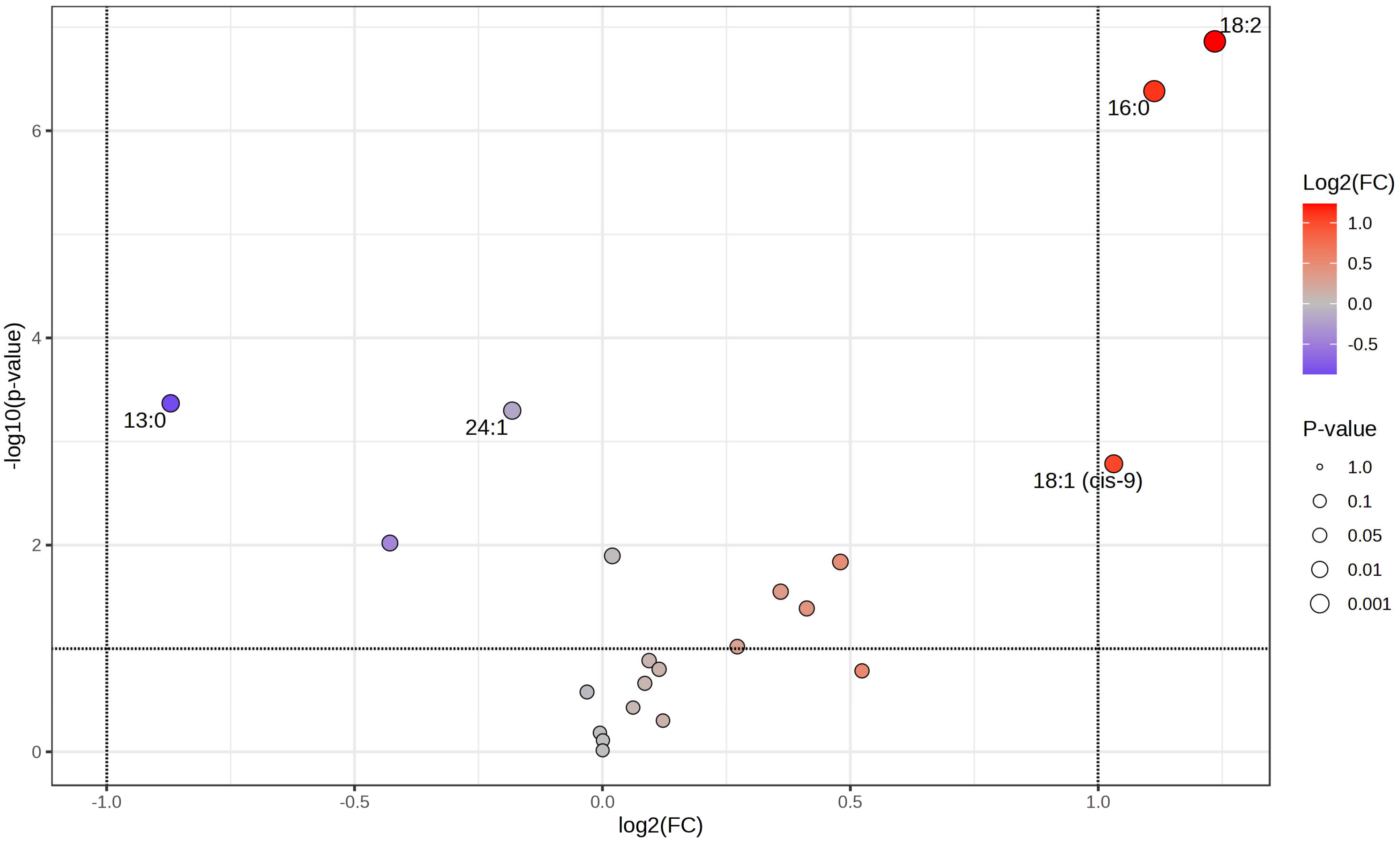

Figure S2. Volcano plot displaying significant differences in fatty acid profiles were development between Alpine and Spanish-Creole goat sperm. The −Log10 was plotted against the log2 (fold change: Alpine and Spanish-Creole); the vertical lines represent ± 1.0-fold change while the hori-zontal line represents the significance threshold ($p<0.05$); Color coding is based on the fold change, blue; down and red; up.
